# Barriers to Cancer Diagnosis and Treatment: A Pilot Qualitative Study of Patient and Practitioner Perspectives in Rural India

**DOI:** 10.7759/cureus.67249

**Published:** 2024-08-19

**Authors:** Akash Nagar, Divya Madamanchi, Gayatri R Nair, Akhil Revikumar, Suman Ray, Sai Mahesh Vajjala, Akhila B S, Shubham Shivale

**Affiliations:** 1 Community Medicine, Dr. D. Y. Patil Medical College Hospital and Research Centre, Dr. D. Y. Patil Vidyapeeth (Deemed to be University), Pune, IND

**Keywords:** barriers, cancer treatment, cultural beliefs, cancer care facilities, health care infrastructure

## Abstract

Introduction

Cancer remains a critical global health issue, particularly in developing countries, where timely diagnosis and effective treatment are often hindered by numerous barriers. These obstacles exacerbate the cancer burden and contribute to disparities in care. This study explores the barriers to cancer diagnosis and treatment from the perspectives of patients and healthcare providers in rural India, aiming to inform targeted interventions and improve outcomes.

Methods

This qualitative study was conducted from April to May 2024 at a tertiary cancer hospital in rural Western Maharashtra, India. Nine semi-structured interviews were conducted with five cancer patients and four healthcare practitioners. Participants were selected through purposive sampling until information saturation was achieved. Interviews were conducted in local languages and analyzed using thematic analysis to identify key barriers and themes.

Results

The study identified several major themes related to barriers to cancer diagnosis and treatment. Patients highlighted a lack of awareness and understanding of cancer, significant financial burdens, challenges in accessing healthcare facilities, and emotional distress. Healthcare practitioners noted systemic issues, including inadequate diagnostic capabilities, insufficient healthcare infrastructure, and a shortage of specialized providers. Both groups emphasized the impact of cultural beliefs and stigma, as well as the limited support systems available to patients.

Conclusion

The findings highlight the complex interplay of factors contributing to delays in cancer diagnosis and treatment in rural India. Addressing these barriers requires multifaceted interventions, including increasing public awareness, improving healthcare infrastructure, and enhancing support systems for patients. Policy development should focus on these areas to reduce disparities and improve cancer care outcomes in resource-limited settings.

## Introduction

Cancer remains one of the most pressing global health challenges of our time, with its impact being particularly severe in developing countries [1]. Despite significant advancements in medical science and technology that have improved cancer outcomes in many parts of the world, numerous barriers continue to hinder timely diagnosis and effective treatment, especially in resource-limited settings. These obstacles contribute to the growing cancer burden and disparities in cancer care across different regions and populations.

Early detection and prompt treatment are crucial factors in improving cancer survival rates and quality of life for patients [2]. However, in many regions, particularly low- and middle-income countries, patients often face significant delays in diagnosis. These delays frequently lead to advanced-stage presentations, which are associated with more complex treatments, poorer prognoses, and increased mortality rates. The reasons for these delays are multifaceted and can be attributed to a complex interplay of patient-related, healthcare provider-related, and health system-related factors [3].

Previous studies have identified various obstacles to early cancer diagnosis and treatment across different regions [4-7]. These barriers can include limited awareness and knowledge about cancer symptoms among the general population, which often results in delayed health-seeking behaviors. Cultural beliefs and practices, such as reliance on traditional medicine or stigma associated with certain cancers, can also play a significant role in deterring individuals from seeking timely medical attention. Economic hardships, including the cost of medical care and loss of income during treatment, present additional challenges for many patients [8-11].

Furthermore, inadequate access to healthcare facilities, particularly in rural or underserved areas, can significantly impede timely diagnosis and treatment [12]. This issue is often exacerbated by a shortage of specialized healthcare providers and advanced diagnostic equipment in many regions. The problem is further compounded by healthcare system challenges such as insufficient diagnostic capabilities, lack of standardized referral pathways, and fragmented or poorly coordinated cancer care services [13,14].

In many developing countries, national cancer control plans are still in their infancy or face significant implementation challenges [15]. While efforts are being made to expand healthcare providers' capacity and training in oncology and related fields, organized screening programs for common cancers are often lacking or inadequately implemented [16]. Additionally, comprehensive cancer care guidelines tailored to local resources and needs are frequently absent or poorly disseminated, leading to inconsistencies in care delivery [17].

The landscape of cancer care is further complicated by the rapid advancements in cancer diagnostics and treatments. While these innovations offer new hope for improved outcomes, they also present challenges in terms of accessibility and affordability, particularly in resource-constrained settings [18]. This creates a widening gap in the standard of care available to patients in different parts of the world.

Given the clinical significance of delayed diagnosis and the limited understanding of region-specific barriers to early cancer detection and treatment, there is an urgent need for comprehensive research in this area. This study aims to examine the obstacles perceived from the perspectives of both patients and healthcare providers across various cancer types. By identifying these barriers, we hope to provide valuable insights that can inform targeted interventions, guide policy development, and ultimately improve cancer outcomes in resource-limited settings.

## Materials and methods

Study design and setting

This study was conducted from April 2024 to May 2024 at a tertiary cancer hospital located in a rural part of Western Maharashtra, India. It encompassed a comprehensive examination of individuals aged 18 years and older attending the radiotherapy and chemotherapy outpatient departments (OPDs) of the hospital.

Ethical considerations

Before the study began, ethical clearance was obtained from the Institute Ethical Committee. Permission was also secured from the institutional review board of Dr. D. Y. Patil Medical College, Hospital and Research Centre, Pune, with approval number I.E.S.C./464/2022. Participants were fully informed about the study's objectives and data collection methods to ensure transparency, and their confidentiality was maintained through a system of codes and numbers. Informed written consent was obtained from each patient or their caregiver before the study commenced.

Eligibility criteria

This research focused on all histopathologically diagnosed cancer patients aged 18 years or older visiting the tertiary cancer hospital, regardless of their gender, caste, or religion, provided they consented to participate in the study. Healthcare professionals who gave consent were also included, while the study excluded patients with diagnosed mental illnesses and patients or practitioners who were unwilling to provide consent.

Data collection and analysis

A total of nine one-on-one semi-structured interviews were conducted with five cancer patients and four healthcare practitioners. Purposive sampling was used to identify participants who were rich in information. The in-depth interviews were conducted using an interview guide in a convenient place and time, in the vernacular languages of English, Hindi, or Marathi, as applicable. Participants were encouraged to express their perceptions freely to gain a deeper understanding of the barriers related to cancer diagnosis and treatment and to suggest ways to overcome them. Interviews were audio recorded with written informed consent from the participants. Each interview lasted between 30 minutes to one hour, with the duration depending on the case. Interviews included greetings and icebreakers as part of a formal introduction, followed by the main conversation. The interviewers also made field notes to capture details of the participants’ nonverbal expressions and the interview context. Information on the patient’s clinical background (gender, age, diagnosis, treatment) was obtained in advance from the attending physician with the participants’ consent. Interviews continued until no new information emerged, indicating data saturation. Participants were given the opportunity to ask questions after the interviews, and their concerns were addressed.

**Figure 1:**
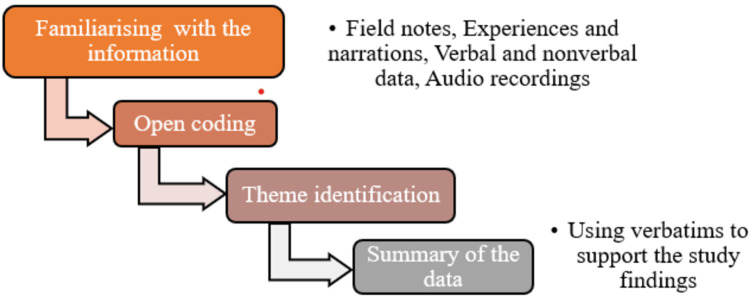
Sequence of analyzing qualitative data This image has been created by the authors.

All nine interviews were transcribed verbatim by a research team member, and the accuracy of the transcriptions was verified against the original recordings by another team member. The qualitative data from healthcare providers and patients were explored through thematic analysis, as illustrated in Figure 1. Thematic saturation was considered achieved when the team determined that no new themes were emerging from the data, indicating a comprehensive capture of the thematic elements present in the interviews.

## Results

**Figure 2:**
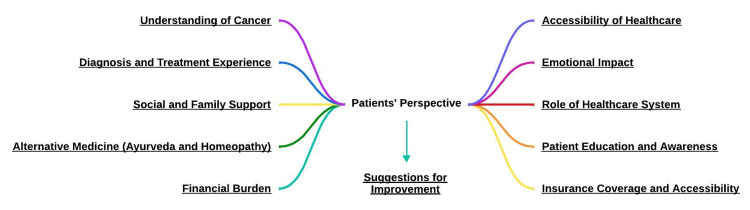
Various themes related to the patient’s perspective on treatment and diagnosis This image has been created by the authors.

**Figure 3:**
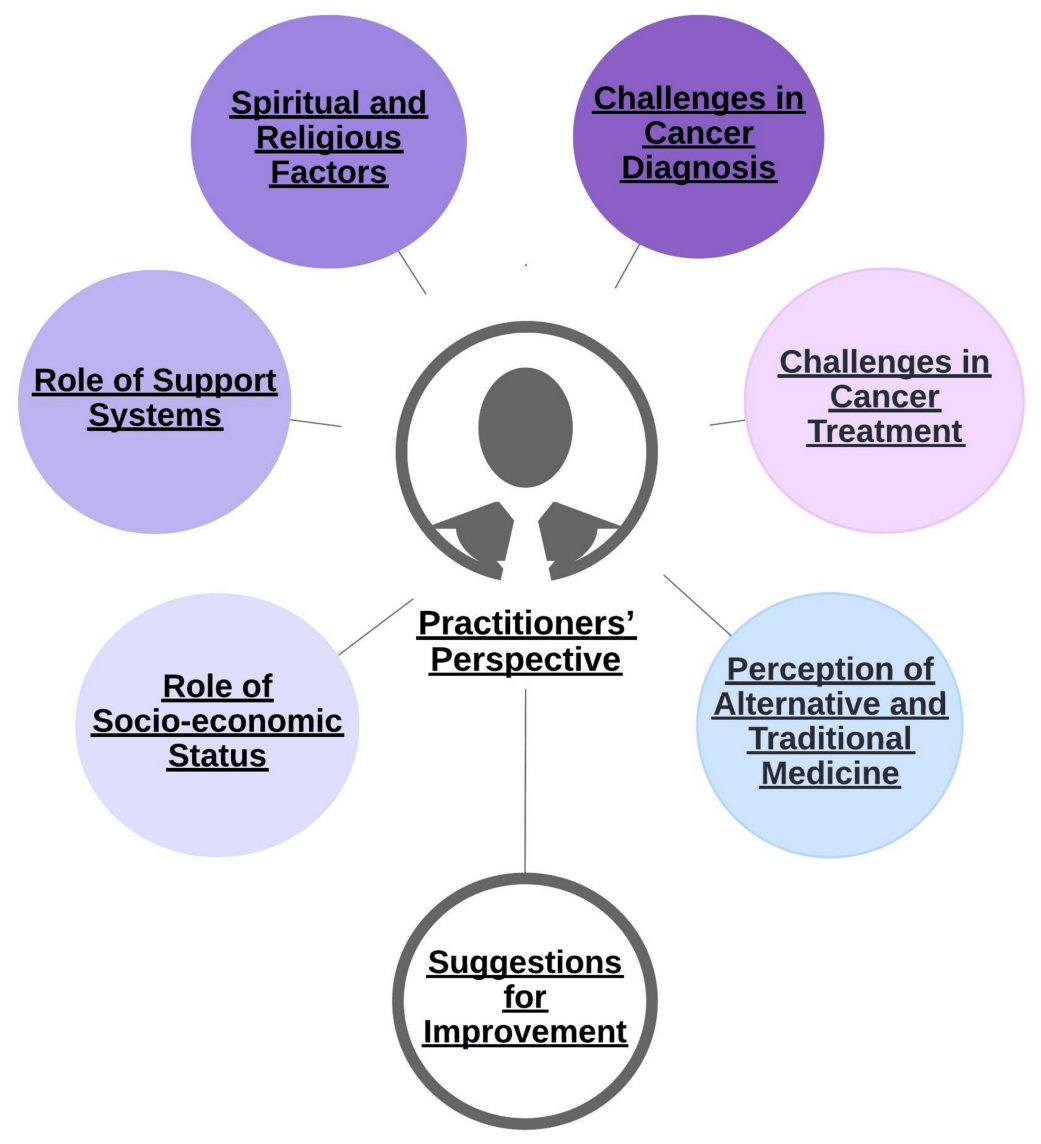
Various themes related to practitioners’ perspective on the treatment and diagnosis of cancer This image has been created by the authors.

We conducted in-depth interviews with five cancer patients (two with lip and oral cavity cancer, one with breast cancer, one with ovary cancer, and one with cholangiocarcinoma), as well as with four practitioners (two palliative care physicians and two oncologists) for our current study. The themes and subthemes, reflecting the perspectives of patients (Figure 2) and practitioners (Figure 3), derived from these interviews and accompanied by relevant verbatim quotes, are detailed below.

Patient's perspective

Understanding of Cancer

This theme explores the depth and accuracy of the patient's knowledge about cancer. It includes their understanding of what cancer is, the perceived causes of the disease, and their beliefs about its curability. The level of understanding can significantly impact their attitudes toward seeking timely diagnosis and adhering to treatment protocols.

Causes of Cancer

Verbatim:

Interview 1: "Cancer is due to the daily diet, pollution, stress, habits, etc."

Interview 1: "Our normal cells convert to cancer cells, and it slowly starts dividing, it starts spreading slowly, it starts damaging the cells of the body, the immunity goes down."

Interview 2: "I don't know; only a few people know how it happens. If they don't know clearly how it happens, how can we know how it happens? I know that smoking leads to cancer."

Interview 4: "It depends on person to person. Some consume something, and it may cause cancer, while there are people who don't consume anything, and they still suffer from cancer."

Interview 5: "Breast cancer occurs because of applying 'Mishri' (a type of tobacco). And secondly, due to eating tobacco. And due to tension. It can also be due to stress."

Curability of Cancer

Verbatim:

Interview 1: "If we get to know in the first or second stage, then there is a treatment. If it increases, then the treatment is not possible, in my opinion."

Interview 2: "Yes, there is a cure. But it depends on your destiny and luck."

Interview 3: "There is a complete cure for cancer; a person should not give up."

Interview 5: "No, it can't be completely cured. When they operated on it for the first time, it was a small tumor. So, the doctor said that we will remove the tumor completely and everything would be all right. He removed it. After six months, the same tumor came back."

Diagnosis and treatment experience

This theme delves into the multifaceted challenges faced by patients during the journey of cancer diagnosis and treatment. It covers the obstacles encountered in recognizing symptoms, accessing healthcare facilities, navigating the complex medical system, and obtaining timely and accurate diagnoses. Furthermore, it examines the physical, emotional, and financial burdens experienced during treatment, including side effects, financial constraints, and logistical difficulties. The interactions and communication with healthcare providers, both in public and private settings, are also explored within this theme.

Challenges in Diagnosis

Verbatim:

Interview 1: "I didn't understand the symptoms. Actually, there was so much pain in the teeth. There was so much pain in the jaw, but I did not show it to the doctor. I did not have money to show it to the doctor, so I took some painkillers on my own. Till the time we went to the doctor, cancer had spread a lot."

Interview 4: "A lot, a lot, a lot of trouble for us. There was a shortage of money, sir. A lot of money was needed just to get diagnosed."

Interview 5: "It took six months to get diagnosed. For investigations, we had to get it done two to three times. For example, computed tomography (CT) scans and positron emission tomography (PET) scans, like this. In that, it costs a lot of money."

Interview 5: "And, people are not aware; people have no idea about the symptoms of cancer."

Challenges in Treatment

Verbatim:

Interview 1: "The private ones provide better treatment, the government one does not. Government hospitals do not pay attention; they just refer directly, and they do not have any facility."

Interview 4: "We had to sell gold. There was no money, nothing. What could we do? We used to wash utensils in people's homes. We have no money left to buy food. We have taken loans for everything, for medicine."

Interview 5: "I am not able to tolerate anything. I am 65 years old. Chemotherapy gives a lot of pain to some patients. It hurts; vomiting also happens. I also had diarrhea and lost also lost my hair."

Experience with Healthcare Providers

Verbatim:

Interview 2: "They talked, but not to me. Now, she is my daughter; they talked to her. They explained everything properly, but there is a little issue with employees' state insurance corporation (ESIC) hospitals, from the beginning itself, there has been some issue with referrals. ESIC people say if there is any compulsion, then only refer; they try to minimize referrals. They said there are some guidelines to develop the in-house facility. But as of now, they do not have a full treatment facility available."

Interview 4: "Yes, he is saying the right thing. I tell them, I tell them, I don't have money, and I am in so much problem, sir. I have taken loans. They (doctors) understand everything and are trying their best."

Interview 5: "In government, they don't pay much attention, and in private, they give attention, they give attention because they need money, and in government, money is less, but don't give much attention."

Social and family support

This theme examines the crucial role of social and family support systems in the lives of cancer patients. It explores the extent of emotional, financial, and practical assistance provided by family members, as well as the presence (or absence) of societal stigma and cultural beliefs surrounding cancer. These factors can significantly influence a patient's willingness to disclose their condition, seek treatment, and cope with the challenges associated with the disease.

Family Support

Verbatim:

Interview 1: "No, nothing. No support! There is no support, means, I have a sister who is married, they come sometimes to see me."

Interview 1: "In the society, there are all types of people. There are rich people, there are also poor people, but no one helped. No one helped. We went to some big influential people, but no one helped."

Interview 4: "My daughter-in-law, my son. Three of us live here. My sister-in-law, my mother-in-law. We are a big family. No one came to see me. No one showed any sympathy, and no one helped."

Interview 5: "There was complete support from home."

Interview 5: "Yes, sir, our patient told us a little late; she told my younger sister that when she took a bath, she told my sister that this is happening in my breast, you see, then my younger sister did a little fast work, to check, to take her to the hospital, that's when everyone came to know."

Social Stigma and Beliefs

Verbatim:

Interview 2: "Someone talks like this; someone talks like that. In the world, it keeps on going."

Interview 3: "Yes, it happens. I have experienced a lot. A lot of people have said a lot of things to us. But I don't believe that. They can say whatever they feel. Their mouth is open. I can't close it. Whatever happened, I did not lose courage."

Interview 5: "I used to roam around with other women. Now, I stopped roaming. I stopped because after the operation, my hair was a little damaged, and the breast was also removed. That's why I was embarrassed. I was embarrassed to meet my friends, that's why I didn't talk to them, and I used to think, that if I tell them, what will they say, how did this happen! That's why I didn't meet them. I was shy and embarrassed."​​​​​​​

Alternative medicine (Ayurveda and Homeopathy)

This theme focuses on the perspectives and experiences of patients and their relatives with alternative medicine approaches, particularly Ayurveda and Homeopathy, in the context of cancer treatment. It explores their beliefs regarding the effectiveness of these alternative therapies, the reasons for choosing or avoiding them, and the outcomes of incorporating them into the treatment regimen.

Perception of Effectiveness

Verbatim:

Interview 1: "Sir, Ayurveda is effective. I have a little knowledge about Ayurveda. According to the stage, if there is cancer in the first stage, then Ayurveda can help and cure it. And same is true for homeopathy. In Ayurveda, it will take a little more time, but both are effective if it is found in the early stage."

Interview 2: "People are saying that Ayurveda also works. The cancer gets reduced with Ayurvedic medicines. But we have not gone anywhere till now."

Interview 3: "60%. You can treat almost 60% of cancer with homeopathy and Ayurveda. I read about it in the newspaper."

Interview 4: "Yes, I used to think it works. We underwent homeopathic treatment, but it didn't work. We went to Karnataka for Ayurvedic treatment. After doing the biopsy, I came here to this private hospital, and then I went to another private hospital. Even there, after chemotherapy, it was not getting reduced. Some people said that if you eat Ayurvedic medicine, it will be reduced very quickly. Then I did it for four to five thousand Rs, it didn't get reduced. Then I came here from there. I started both of them, Ayurvedic and chemotherapy, but nothing happened."​​​​​​​

Financial burden

This theme highlights the significant financial strain and economic hardships faced by patients and their families during the cancer diagnosis and treatment process. It encompasses the high costs associated with various medical investigations, surgical procedures, chemotherapy, radiation therapy, and other treatments. Additionally, it sheds light on the lack of adequate financial support mechanisms, such as comprehensive insurance coverage or government assistance, which can exacerbate the financial burden. The theme also explores the coping strategies employed by patients and their families, such as taking loans, selling assets, or seeking financial aid from others to meet the mounting expenses associated with cancer care.

Cost of Diagnosis and Treatment

Verbatim:

Interview 1: "He was asking for 3.5 lakhs." (referring to the high cost quoted by a doctor)

Interview 4: "We had to sell gold. There was no money, nothing. What could we do? We used to wash utensils in people's homes. We have no money left to buy food. We have taken loans for everything, for medicine."

Interview 4: "I gave Rs. 12,000 and did a biopsy in pandemic, that's when I came to know. A lot, a lot, a lot of trouble for us. There was a shortage of money, sir. A lot of money was needed just to get diagnosed."

Interview 5: "For investigations, we had to get it done 2-3 times. For example, CT scans, PET scans, like this. In that, it costs a lot of money."

Financial Support

Verbatim:

Interview 1: "No, but she has a card in the Mahatma Jyotirao Phule Jan Arogya Yojana (MJPJAY) scheme as she has a billow poverty line (BPL) card. The scheme has covered expenses of surgery, which was around 60,000 Rs."

Interview 2: "It is in the Employees' State Insurance Scheme (ESIS). It is (treatment) not fully covered though."

Accessibility of healthcare

This theme explores the barriers and challenges that patients encounter in accessing appropriate and timely healthcare services for cancer diagnosis and treatment. It encompasses geographical barriers, such as the distance from healthcare facilities, particularly in rural or remote areas, and the lack of nearby specialized cancer treatment centers. Additionally, it highlights the long waiting times and delays experienced by patients in receiving diagnostic tests, consultations, and initiating treatment, often due to resource constraints or an overwhelming patient load. The theme also touches upon the logistical and practical difficulties faced by patients in navigating the healthcare system, such as obtaining referrals, coordinating appointments, and transferring between different healthcare providers or institutions.

Geographical Barriers

Verbatim:

Interview 5: "Sir, still, it took six months. Sir, because we were in Khedegaon (a village), there was no hospital nearby; that's why we had to go to the city, there also they didn't check properly and same investigation were done once, twice, three times..."​​​​​​​

Long Waiting Times

Verbatim:

Interview 1: "They said that she has to wait for four to five months as the waiting list is very long."

Interview 1: "It takes at least 15 days to get tests done."

Emotional impact

This theme delves into the profound psychological and emotional toll that a cancer diagnosis and its subsequent treatment can have on patients and their loved ones. It examines the distress, anxiety, fear, and uncertainty that patients may experience throughout their journey, including the fear of the unknown, the potential side effects of treatment, and the uncertainty surrounding treatment outcomes. Additionally, the theme explores the psychological impact of physical changes, such as hair loss or surgical disfigurement, on the patient's self-esteem and emotional well-being. Furthermore, it highlights the potential for denial or avoidance as coping mechanisms, which can delay seeking timely medical attention and treatment.

Psychological Distress

Verbatim:

Interview 2: "I don't want to say anything. I don't understand. I don't know anything. I have heard of it. I used to be afraid of even taking the name of cancer. But now it is in my destiny."

Interview 5: "The patient is not able to tolerate anything. Chemotherapy gives a lot of pain to some patients. It hurts, vomiting also happens, and the patient was 65 years old. He was also affected by diarrhea, and she also lost her hair."​​​​​​​

Fear and Denial

Verbatim:

Interview 1: "They do not get it done out of fear. They do not know about the side effects, what are the side effects, what are the benefits of treatment."

Interview 5: "So, the doctor said that we will remove the tum0r completely and everything will be all right. He removed it. After six months, the same tumor came back. Yes, gradually, it started to grow again."

Role of the healthcare system

This theme focuses on the critical role played by the healthcare system in providing comprehensive and effective care for cancer patients. It highlights the challenges posed by the lack of resources and facilities, particularly in government hospitals, which can limit access to specialized cancer care and advanced treatment options. Additionally, the theme explores issues related to referrals and coordination between different healthcare providers and institutions, which can impact the continuity of care and lead to delays or duplication of diagnostic tests and procedures. Furthermore, it addresses the need for streamlined processes, clear communication, and efficient coordination among healthcare professionals to ensure a smooth and seamless patient experience.

Lack of Resources and Facilities

Verbatim:

Interview 1: "There is a limit of the test that can be done, and number of blood samples taken in a day and also they did not understand the symptoms."

Interview 2: "And in the government hospital also, there should be treatment facilities. Like there was no facility in ESIC so we had to come here. If there was a facility in ESIC, then we didn't have to come here."

Referral and Coordination Issues

Verbatim:

Interview 1: "Although she had multiple biopsies done at different hospitals, which was a problem, and also, since we go referred from two to three places, its added to the overall expenses and took time."

Interview 2: "It is difficult to give a referral for now, it’s a little difficult for ESIC hospitals to give a private hospital referral from the first of this month."

Patient education and awareness

This theme emphasizes the pivotal role of patient education and public awareness in facilitating early detection, timely diagnosis, and appropriate treatment for cancer. It explores the lack of knowledge and awareness about cancer symptoms among patients, which can lead to delays in seeking medical attention and ultimately impact the effectiveness of treatment. The theme also highlights the need for targeted awareness campaigns and educational initiatives aimed at empowering patients and their families with accurate information about cancer, its risk factors, and the importance of regular screening and preventive measures. Additionally, it underscores the importance of open communication between healthcare providers and patients, enabling patients to make informed decisions and actively participate in their treatment journey.

Lack of Knowledge About Symptoms

Verbatim:

Interview 1: "She didn't go to the doctor. She took painkillers for two months. After that, she developed a node in mouth. Then we went to the central hospital in Thane district."

Interview 1: "If I had shown it to the doctor, they would have asked for 300-500 Rs as fees. We did not have that much money. Till the time I went to the doctor, cancer had spread a lot."

Interview 2: "If we knew, we wouldn't have stayed at home. I would have consulted quickly."

Need for Awareness Campaigns

Verbatim:

Interview 1: "People should be made more aware about early symptoms, so if they already know about this, they will come for treatment soon."

Interview 4: "So, the government should spread more awareness about cancer."

Insurance coverage and accessibility

This theme explores the availability, limitations, and challenges associated with insurance coverage for cancer treatment. It highlights the existence of various government and private insurance schemes but also underscores the limitations and restrictions that patients may face in fully availing the benefits of these schemes. Additionally, it sheds light on the difficulties patients encounter in navigating the bureaucratic processes and meeting the eligibility criteria for insurance coverage. Furthermore, the theme emphasizes the need for comprehensive and streamlined insurance schemes that can provide financial support to patients throughout their treatment journey without imposing unnecessary barriers or out-of-pocket expenses.

Subtheme: Availability and Limitations of Insurance Plans

Verbatim:

Interview 2: "It is in Employees' State Insurance Scheme (ESIS). It is not fully covered though."

Interview 3: "There is an insurance, Rajiv Gandhi Scheme, treatment is fully covered under it. When the operation was going to happen, our Rajiv Gandhi insurance got canceled. So, we were not getting insurance. We had to spend for the operation from our side."​​​​​​​

Suggestions for improvement

This theme encompasses the recommendations and suggestions provided by the patients and their relatives to enhance various aspects of cancer care and support. It includes their insights on increasing public awareness and education about cancer symptoms and the importance of early detection. Additionally, it covers proposed improvements in healthcare systems, such as streamlining government schemes, enhancing facilities, and fostering open communication between healthcare providers and patients. Furthermore, it highlights the need for emotional and financial support mechanisms, particularly for underprivileged patients, to ensure adherence to treatment and alleviate the associated burdens.

Awareness and Education

Verbatim:

Interview 1: "People should be made more aware about early symptoms, so if they already know about this, they will come for treatment soon."

Interview 5: "Look, sir, now this cancer, earlier it was not such a big disease, but now since 2018 it is spreading more, it is spreading more in ladies, and the educated people, they know about cancer, but people in villages have very less idea about cancer."​​​​​​​

Healthcare System Improvements

Verbatim:

Interview 1: "In the society, there are all types of people. There are rich people, there are also poor people, but no one helped. No one helped. We went to some big influential people, but no one helped."

Interview 2: "The government should also support. Schemes should be streamlined as it is very difficult to avail government schemes. And in the government hospital also, there should be treatment facilities. Like there was no facility in ESIC hospitals.”

Interview 5: "Look, first thing is that whatever disease the patient has, he should inform the doctor without any shame, and the doctor should also give him complete information and treat him."

Practitioners’ perspective

Challenges in Cancer Diagnosis

This theme covers the various obstacles and barriers faced in the timely diagnosis of cancer. It includes subthemes such as delayed diagnosis due to lack of awareness or specific symptoms, cultural beliefs and stigma surrounding cancer, and lack of screening and diagnostic facilities, especially in rural areas.

Delayed Diagnosis

Verbatim:

Interview 1: "Delayed diagnosis! How many in the sense, out of total we see 10 cases per day, delayed diagnosis will be in almost five to six cases, around 50%."

Interview 3: "Most of the patients because carcinoma ovary and all those cases used to detect very late due to very unspecific symptoms. Patient used to present very late usually in stage 3 and stage 4."​​​​​​​

Lack of Awareness and Health Literacy

Verbatim:

Interview 1: "First thing is, in rural setup, these patients, they don't go to a proper educated practitioner. They tend to go to some quacks, or some Ayurvedic specialists, who doesn't even know what cancer is, who doesn't even know what a tumor looks like."

Interview 2: "Health literacy is very less in India. So, people try to ignore it. All symptoms are coming, but still, they will ignore. Females, they are having back pain and all. Then they will come to hospital. I am having low back pain. They will hide. There was a lump in the breast. They won't tell family members."

Interview 4: "Lack of awareness. And in females, maybe CA cervix and CA ovary everything will start like maybe five years before they come to the OPD. They must be having recurrent urinary tract infections (UTIs). They must be having gastritis and different issues. And they feel like it is normal."​​​​​​​

Cultural Beliefs and Stigma

Verbatim:

Interview 1: "No, I have never encountered cultural stigma. Once they get diagnosed, we will talk about treatment, cultural things that can play a role. Of course, only counselling works."

Interview 2: "Usually, the population who come to the hospital are mostly less educated group, very low-income family. What to say? They may be truck drivers, tempo drivers, shopkeepers. They have the habit. The culture in that side where I work is that they have a tradition of having paan (betel leaf), tobacco. It is a tradition. For them, it is nothing wrong. Nothing wrong."

Interview 3: "There are lot of cultural beliefs playing a role in this. Because in some cultures some of things are not, what do I say, like females do not talk about all these things."

Lack of Screening and Diagnostic Facilities

Verbatim:

Interview 3: "Then among males, this prostate cancer and everything. Always people will neglect those cases. And coming to CA oral cavity, usually it will be present at the earlier stage because it is oral cavity. And many other issues, maybe some other issues like meds or something. People think like it is due to some other reason."

Interview 4: "Primary care centers or grass root centers, anyway you can't diagnose or treat due to lack of facility."​​​​​​​

Challenges in cancer treatment

This theme encompasses the difficulties and challenges encountered during the treatment of cancer patients. It includes subthemes like treatment dropouts and non-compliance, financial barriers that limit access to treatment, lack of infrastructure and resources for optimal treatment, and communication and counseling challenges faced by healthcare providers.

Treatment Dropout and Non-compliance

Verbatim:

Interview 1: "In my practice in a year, if at all I see some around 100 patients, there will be some patients of around 10 to 15 who can drop out of the treatment. And they will shift to Ayurveda in between."

Interview 3: "They will deny themselves. They are like, we don't want the treatment."​​​​​​​

Financial Barriers

Verbatim:

Interview 1: "So, financial aspect is one factor where private patients have a advantage, but whoever is undergoing cancer treatment if at all is able to spend money, they won't mind about spending money. They just want a good treatment."

Interview 3: "And financial issue is there anyway. And one question arises, should I do the treatment of my mother (family), or should I feed my children? Or should I send them to school? So, the moral dilemma is there."

Lack of Infrastructure and Resources

Verbatim:

Interview 3: "Treatment modalities are good. But see, if at all a person should start a private institution with whole oncology set up in India, it will cost you around 40 to 50 crores. So, once you invest 40 to 50 crores of money and you are expecting some revenue out of that. Then these government schemes or welfare schemes. These things don't serve the purpose."

Interview 3: "It is not at par with Western treatment, mostly because of very stringent laws and everything. Clinical trials are happening more in a better way (in Western countries). Research is better. And in India, it is very difficult, and it is very difficult to enroll people for cancer research, which is an important part of developing new drugs."

Communication and Counseling Challenges

Verbatim:

Interview 1: "If they are counseled well, they generally don't tend to leave. Counseling anyways, every doctor should do the counseling. After doing the counseling, also patients will go on. So, those patients, we cannot force them to take the treatment."

Interview 3: "Once the patient is diagnosed, first I will talk with the bystanders, relatives. I don't disclose to the patient directly. First thing. Once the bystanders or relatives are comfortable only, with their permission, I will disclose everything to the patient. But without disclosing to the patient, I will never start my treatment."

Interview 4: "And sometimes what happens is like some people want to talk a lot about their problems and everything. But in rounds, we can give as much as time we want, and in hospital also, we can give as much as time we want. But in outpatient department (OPD), it is sometimes difficult to listen to all their problems because of the rush, way too much, way too many patients."

Perception of alternative and traditional medicine

This theme explores the practitioners' perspectives on the use of alternative and traditional medicine for cancer treatment. It includes subthemes related to the reasons for patients dropping out of conventional treatment and opting for alternative therapies, as well as the perceived effectiveness (or lack thereof) of these alternative approaches.

Use of Alternative Medicine and Dropout from Conventional Treatment

Verbatim:

Interview 1: "Emotional problem only, that's it. Side effects related. And community, there is some people will say Ayurveda will be working; some people will say allopathic is working. So, we cannot force any patient to take our treatment, right. It's up to the patient to take whatever treatment he or she wants."

Interview 3: "Not just homeopathy, Ayurveda. You can't just say it is exactly Ayurveda or homeopathy, but they are some people. Some practitioners on local traditional medicines also but some of them are actual practitioners of Indian System of Medicine (ISM), and they do this acupuncture treatments and Hijama (cupping therapy) treatments."

Perception of Effectiveness of Alternative Medicine

Verbatim:

Interview 1: "Not proven scientifically. But people say it's been working but it's not scientifically proven. Even these people think that the tumor bleeds and the cancer is cured. So, they think that the tumor started bleeding; the waste blood is coming out. So, that's what I encountered with some patients. They say, sir, I am bleeding, bad blood came out, I got cancer treatment from Ayurveda."

Interview 4: "Okay. So, many people get attracted to that, they leave the treatment in between and they go for that. There are many famous people, many famous actors also who went to this alternate path and died very young."

Role of socioeconomic status

This theme highlights the impact of socioeconomic status on access to cancer treatment and the quality of care received. It includes subthemes related to the disparities in treatment access and quality based on socioeconomic status, as well as the financial burden on patients from different economic backgrounds.

Impact on Treatment Access and Quality

Verbatim:

Interview 1: "For private patient, I will be using a different suture meter. For the government patient, I will be using low-quality suture material. And for private, I will be using good medicines. For the government patient, we will be using the grade 2, grade 3 medicines."

Interview 1: "One is obviously socioeconomic status of the patient. Other than that, the Stage of the disease. Stage of the disease definitely will affect the outcome."

Financial Burden on Patients

Verbatim:

Interview 3: "No, because sometimes treatment is not based on the socioeconomic status basically, it is based on the stages. If you are like high socioeconomic status or low socioeconomic treatment is the same because chemotherapy drugs are same."

Role of support systems

This theme explores the importance of support systems for cancer patients, including emotional and psychological support, as well as the role of family and caregivers. It highlights the need for comprehensive support to improve treatment outcomes.

Emotional and Psychological Support

Verbatim:

Interview 2: "There were psychologists also. But if psychologists are not there, we used to counsel. In special cases. Mainly pediatric cases. Relatives need doctors. They don't want to talk to anyone else. They won't believe anyone else."

Interview 3: "Good support is needed for the patient, not just financially, emotionally, and psychologically, spiritually, everything is needed. So, if all those things are provided properly, I think the outcome of cancer treatment will be high."

Role of Family and Caregivers

Verbatim:

Interview 2: "There is another feeling of dependability. Elderly people don't tell their children as they (the elderly) are dependent on them. So, there will be an extra burden for their children. There will be a problem for them. Their children don't know anything."

Interview 3: "Even if some patient is having some cancer or something, you can't inform their local ASHA worker because it's a breach of confidentiality. Even if you want to follow up, you can't do that."

Spiritual and religious factors

This theme delves into the influence of spiritual and religious beliefs and practices on cancer diagnosis and treatment. It includes subthemes related to specific religious beliefs and practices that may impact cancer care, as well as the role of spirituality in coping with the disease.

Religious Beliefs and Practices

Verbatim:

Interview 1: "In some Muslim patients, I do encounter referring cancers as male cancers and female cancers, they call it. I don't know what their cultural background behind that is. They differentiate cancer with gender. Male means they think it's aggressive cancer and female cancer is weak cancer, like that they call it. There are no such terminologies, I used to tell them."

Interview 3: "And then some religious people, if you think about some infectious sources of cancers, there are some religions that leads to some practices that lead to these issues. Cancers, I mean."

Interview 4: "And sometimes, religious issues are there. As in like, some people are very adamant about... See, blood transfusion if you need it, you have to get a transfusion. And some people don't like people who are lower than their caste to come and treat them."

Spirituality and Coping

Verbatim:

Interview 3: "So, some people become like, some other people become very spiritual, and they started to like, they will start praying early in the morning and going to temple, churches, mosques, and all those things. So, that helps in providing support to the patient."

Interview 3: "And religion-based non-government organizations (NGOs) are also there. So, those people will help you. If you are religious and you are willing to do more to the religion. Like, if you are ready to visit temples, churches, mosques."

Suggestions for improvement

This theme covers the suggestions and recommendations provided by practitioners for improving cancer diagnosis and treatment. It includes subthemes focusing on increasing awareness and health literacy, strengthening screening and diagnostic facilities, and improving infrastructure and resources for cancer care.

Increasing Awareness and Health Literacy

Verbatim:

Interview 2: "First of all, patients, they don't know what cancer, or its symptoms is. They don't know what cancer symptoms can be. They are having a grittiness sensation on the cheeks. But they don't feel like they should go for a checkup if, they feel that it is normal. They feel pain in the teeth. But they won't go for a checkup if something is happening in the jaw or bone. They don't have this feeling."

Interview 3: "Improving awareness, infrastructure, funding, then more specialists, more doctors, nurses, and paramedical staff. These things are the most important."

Strengthening Screening and Diagnostic Facilities

Verbatim:

Interview 1: "I feel that all the rural centers, all the primary health centers, they should have a separate cancer division, Primary, for diagnosis purposes. Like, taking an initial biopsy, getting a basic chest X-ray done, or tumor markers and blood tests facilities. The specific kit for diagnosing cancer can be made."

Interview 3: "Strengthen district hospital, the proper hierarchy of transfer of cases, grassroots level designated individuals for preventive check-ups and regular check-ups and improve the awareness among females regarding this hard-to-find cancers, regarding these non-specific symptoms."

Improving Infrastructure and Resources

Verbatim:

Interview 1: "Here, the problem comes with only government scheme welfare patients. Insurance patients and private patients, they all get good quality treatment only. Whatever they have wanted, we can provide it. We have the infrastructure, we have the quality, we have those surgeons, we have those medicines also. Basically, the poorest of the poor gets the brunt of the problem."

Interview 2: "It is always better to be cautious because cancer is fatal. So, it is better to do some test if it is warranted rather than under-diagnosing and thinking the constant headache that is because of migraine and actually being a cancer of lung with a metastasis to brain or cancer of ovary or breast."

Interview 4: “The health gross domestic product (GDP) itself, the health budget itself is lowest every year. So, once they give to the health sector, only then we can give quality treatment to our patients.”

## Discussion

A study conducted by Kumar et al. [9] in Bangalore, India, identified that the reasons for presentation delay included misconceptions about cancer, stigma, social responsibilities, and fear of diagnosis, while treatment delays were attributed to factors such as cost, distance, perceptions about treatment, and dissatisfaction with public health services. Suggestions to reduce these delays focused on improving awareness, training private providers, and addressing health system barriers. Similar themes emerged in the current study. A lack of comprehensive understanding of cancer among patients and their relatives was noted, with beliefs ranging from lifestyle factors to destiny impacting timely treatment and adherence. Diagnosis and treatment experiences revealed challenges such as delays due to symptom misinterpretation, financial constraints, and inadequate healthcare access. The treatment process was physically and emotionally taxing, with mixed experiences regarding healthcare provider support. Family support varied, with some patients lacking emotional and financial assistance, and social stigma and cultural beliefs influenced the willingness to disclose the condition and seek treatment. Perceptions about alternative medicine like Ayurveda and Homeopathy varied, with some believing in their effectiveness and others having no success or not exploring them. The high costs of cancer diagnosis and treatment led to significant financial strain, with many patients taking loans, selling assets, or seeking financial aid due to insufficient insurance coverage or government assistance. Geographic barriers and long waiting times hindered access to timely care, compounded by resource constraints and overwhelming patient loads. The emotional impact on patients and their loved ones was profound, with significant psychological distress, anxiety, and fear often leading to delays in seeking medical attention. Challenges within the healthcare system included resource limitations, lack of specialized care, and issues with referrals and continuity of care, leading to delays and treatment disruptions. A lack of knowledge about cancer symptoms delayed medical attention, highlighting the need for targeted awareness campaigns to empower patients with accurate information. Insurance coverage issues, such as partial coverage, cancellations, and bureaucratic hurdles, further complicated the treatment process, necessitating comprehensive and streamlined insurance schemes to alleviate financial strain. Finally, suggestions for improvement included increasing public awareness, enhancing healthcare facilities, improving communication between doctors and patients, and providing emotional and financial support to improve cancer care. Comparable results were seen in another study by Zammit et al. [13].

The current pilot study also explored practitioners' perceptions regarding cancer diagnosis and treatment, revealing several major themes. Practitioners highlighted significant challenges in timely cancer diagnosis, citing delayed diagnoses due to lack of awareness, cultural stigma, and inadequate screening facilities, especially in rural areas. These findings were similar to that of the studies conducted before [11,19]. Treatment challenges included high dropout rates and non-compliance due to financial constraints, severe side effects, and a preference for alternative therapies. Financial barriers were exacerbated by limited resources and infrastructure, with treatment quality sometimes compromised. Practitioners had mixed views on alternative medicine, recognizing patient autonomy but noting a lack of scientific evidence for these treatments. Spiritual and religious beliefs also influenced patients' approaches to cancer, with some beliefs affecting diagnosis and treatment decisions. Socioeconomic status played a crucial role in accessing quality care, with lower-income patients facing significant disparities. The importance of support systems, including psychological and family support, was emphasized. Practitioners suggested increasing awareness and health literacy, enhancing screening and diagnostic facilities, improving infrastructure and resources, and ensuring equitable access to quality treatment for all socioeconomic groups.

Reducing the diagnostic interval could result in patients coming to medical attention earlier, potentially improving outcomes. Our study reflects that knowledge alone is insufficient for promoting timely help-seeking. It is, therefore, crucial to address barriers to accessing medical care, and here, efforts at both the patient and provider levels are required. The study findings have important implications for clinical practice, policy, and public health interventions aimed at reducing diagnostic delays in cancer. Strategies to address disparities in healthcare access, improve health literacy, and enhance awareness of cancer symptoms are essential for facilitating early detection and timely diagnosis. Additionally, initiatives to strengthen primary care, streamline referral pathways, and expedite diagnostic testing are crucial for minimizing delays and improving patient outcomes. Efforts to improve health education and awareness among this demographic group are vital for facilitating early detection and prompt referral for diagnostic evaluation.

Limitations

Like any other study, this one, too, had certain limitations. With a small sample size, the findings may not be representative of the broader rural Indian population, limiting their generalizability. Participants' recollections may be affected by recall bias, especially given the emotional and psychological toll of their experiences. While the study acknowledges the role of cultural beliefs and stigma, a more thorough exploration of specific cultural practices across different communities could offer a more comprehensive understanding. Additionally, while interviews were conducted in local languages, efforts were made to transcribe them as closely as possible to the original statements made by the participants in an effort to ensure accurate translation. Consequently, some quotes may contain grammatical errors, while some nuances may have been lost or misinterpreted.

## Conclusions

This pilot study provides valuable insights into the complex landscape of cancer diagnosis and treatment in rural India, revealing a multitude of interconnected barriers faced by both patients and healthcare providers. The findings highlight significant challenges, including delayed diagnosis due to lack of awareness, cultural stigma, inadequate screening facilities, financial constraints limiting access to quality care, and systemic issues within the healthcare infrastructure.

The study highlights the need for a multifaceted approach to improve cancer care in resource-limited settings. Key areas for intervention include enhancing public awareness and health literacy, strengthening primary care and referral systems, improving access to diagnostic facilities, and addressing socioeconomic disparities in healthcare access. Additionally, the role of family support, cultural beliefs, and alternative medicine practices emerged as important factors influencing patient decisions and treatment outcomes. Further research with a larger sample size is necessary to validate these findings and explore innovative strategies to mitigate these barriers.
